# Effects of cold plasma, gamma and e-beam irradiations on reduction of fungal colony forming unit levels in medical cannabis inflorescences

**DOI:** 10.1186/s42238-020-00020-6

**Published:** 2020-02-28

**Authors:** Shachar Jerushalmi, Marcel Maymon, Aviv Dombrovsky, Stanley Freeman

**Affiliations:** 1 Department of Plant Pathology and Weed Research, The Volcani Center, Agriculture Research Organization, 7505101 Rishon Lezion, Israel; 2 The Robert H. Smith Faculty of Agriculture, Food and Environment, The Hebrew University of Jerusalem, 7610001 Rehovot, Israel

**Keywords:** *Botrytis cinerea*, CFU, Cold plasma, E-beam, Gamma irradiation, Medical Cannabis, Sterilization

## Abstract

**Background:**

The use of medical cannabis (MC) in the medical field has been expanding over the last decade, as more therapeutic beneficial properties of MC are discovered, ranging from general analgesics to anti-inflammatory and anti-bacterial treatments. Together with the intensified utilization of MC, concerns regarding the safety of usage, especially in immunocompromised patients, have arisen. Similar to other plants, MC may be infected by fungal plant pathogens (molds) that sporulate in the tissues while other fungal spores (nonpathogenic) may be present at high concentrations in MC inflorescences, causing a health hazard when inhaled. Since MC is not grown under sterile conditions, it is crucial to evaluate current available methods for reduction of molds in inflorescences that will not damage the active compounds. Three different sterilization methods of inflorescences were examined in this research; gamma irradiation, beta irradiation (e-beam) and cold plasma to determine their efficacy in reduction of fungal colony forming units (CFUs) in vivo.

**Methods:**

The examined methods were evaluated for decontamination of both uninoculated and artificially inoculated *Botrytis cinerea* MC inflorescences, by assessing total yeast and mold (TYM) CFU levels per g plant tissue. In addition, e-beam treatment was also tested on naturally infected commercial MC inflorescences.

**Results:**

All tested methods significantly reduced TYM CFUs at the tested dosages. Gamma irradiation reduced CFU levels by approximately 6- and 4.5-log fold, in uninoculated and artificially inoculated *B. cinerea* MC inflorescences, respectively. The effective dosage for elimination of 50% (ED_50_)TYM CFU of uninoculated MC inflorescence treated with e-beam was calculated as 3.6 KGy. In naturally infected commercial MC inflorescences, e-beam treatments reduced TYM CFU levels by approximately 5-log-fold. A 10 min exposure to cold plasma treatment resulted in 5-log-fold reduction in TYM CFU levels in both uninoculated and artificially inoculated *B. cinerea* MC inflorescences.

**Conclusions:**

Although gamma irradiation was very effective in reducing TYM CFU levels, it is the most expensive and complicated method for MC sterilization. Both e-beam and cold plasma treatments have greater potential since they are cheaper and simpler to apply, and are equally effective for MC sterilization.

## Background

In recent years there is a growing trend of research regarding the beneficial effects of medical cannabis (MC) for treating various diseases and ailments (Ben Amar 2006; Ruchlemer et al. 2015). The use of MC is growing exponentially, especially in patients suffering from different types of cancer and HIV, following FDA approval (Ruchlemer et al. 2015), MC is also used widely as a general analgesic. Since MC is used by patients with a weakened immune system, there are potential risks to their health when exposed to microbial-infected cannabis (fungal spores, bacteria, etc.), as shown by a growing number of reports (Cescon et al. 2008; Gargani et al. 2011; Hazekamp 2016; Ruchlemer et al. 2015). Therefore, it is critical to supply MC-treated patients with a “clean”, mold-free and healthy product.

In order to achieve a high level of quality control, many countries, including Israel, the Netherlands, and the European pharmacopoeia have imposed strict regulations dictating the permitted number of microbial contaminations present in commercial MC supplied to patients as, 2000, 100 and 50,000 colony forming units (CFUs) of total yeasts and molds (TYM) per g inflorescence, respectively (https://www.health.gov.il/hozer/mmk151_2016.pdf, EP 8.0, 5.1.8.C) (Hazekamp 2016). These CFU limitations are very low and to date, effective cultivation of MC under sterile conditions does not exist. Therefore, the need for post-harvest decontamination of inhaled MC products is essential. Even though sterilization methods such as autoclaving or ultraviolet (U.V.) irradiation may be the first to come to mind, the most important therapeutic compounds in MC (cannabinoids and terpenes) are heat and light sensitive and undergo decarboxylation, causing their early decay when exposed to the above decontamination methods (Hazekamp 2016; Russo 2011; Small 2016). This highlights the necessity for novel techniques to disinfect MC without exposing the product to high temperatures or U.V. irradiation.

Both gamma and beta radiation (e-beam) fall under the category of ionizing radiation, containing an amount of energy that causes excitation or ionization of atoms and molecules, leading to the creation of free radicals (Jeong et al. 2015). These free radicals in turn sever certain chemical bonds that lead to damage of molecules and especially cell DNA. Damage in these cases can be either direct, caused by Reactive Oxygen Species (ROS) created from the radiolysis of H_2_O_2_, or indirect, caused by other free radicals (Sádecká 2007). In living organisms, these damaged molecules cause a disruption in the chemical and metabolic functions of living cells thus leading to cell death (Hazekamp 2016; Jeong et al. 2015; Sádecká 2007). The advantages of using gamma and beta irradiation for MC decontamination are numerous. Ionizing radiation leaves no residues after application (unlike fungicides for example) and does not involve extreme heat or U.V. irradiation which may damage the active compounds in MC (Hazekamp 2016). Studies related to the effects of gamma and beta irradiation on decontamination of the final product of MC is limited, however, these treatments do not appear to have a detrimental effect on the quality of food and spice products (Arvanitoyannis et al. 2009; Guerreiro et al. 2016; Jeong et al. 2015; Sádecká 2007).

Gamma irradiation is commonly based on the use of cobalt 60 isotope (^60^Co) which is reported as safe for decontamination of both MC and various food products (Arvanitoyannis et al. 2009; Jeong et al. 2015; Sádecká 2007). Moreover, long term mammalian studies have shown that irradiated foods are both safe and nutritious for human consumption (Thayer et al. 1996). While gamma irradiation is more commonly used, e-beam is a newer method showing greater promise. This technique does not require a radioactive source as the radiation is created using an electron accelerator making it environmentally friendly. Moreover, it was reported that a similar efficacy of decontamination was observed when *Botrytis cinerea* (a major MC inflorescence fungal pathogen) was exposed to either gamma or beta irradiation (McPartland et al. 2017). Furthermore, another fungal pathogen *Penicillium expansum*, was more sensitive to e-beam than gamma irradiation (Jeong et al. 2015). While there was no direct mention of *P. expansum* as a specific phytopathogen of MC, *Penicillium* spp. spores are ubiquitous and common in dry MC products, suggesting that this fungus may be a potential pathogen of concern (McPartland et al. 2017; Punja et al. 2019).

While gamma and e-beam irradiation possess a similar mode of action, cold plasma treatment is a different method for sanitation and sterilization. The general definition of plasma is a state of ionized gas, with limited net charge. Natural examples of plasma are the sun and the aurora (Misra et al. 2019; Turner 2016). Cold plasma is usually achieved by deploying electrical discharges in gases at atmospheric or subatmospheric pressure. When a high enough voltage is reached a breakdown of the gas occurs, leading to the formation of a mix of antimicrobial elements. The mechanisms that take place during this phase of cold plasma reaction are numerous and include vibration and excitation of gas atoms, ion-ion neutralization, quenching and many more (Misra et al. 2019; Sahu et al. 2017). Addition of H_2_O_2_ to the plasma, augments the sterilization mechanism; e.g. it was shown that at a high concentration, ROS inhibit cell proliferation and cause apoptosis (Thannickal and Fanburg 2000). Many reports have reported the efficacy of cold plasma treatment in inactivating a wide spectrum of bacteria (gram positive and negative) and in many of these studies the method was shown to be even more effective in the reduction of fungal viability and spore CFU counts (Hertwig et al. 2015a, 2015b; Kim et al. 2014; Misra et al. 2019; Zahoranová et al. 2016). In recent years cold plasma sterilization has become more popular in various medical applications such as surface sterilization and sterilization of damaged tissues (Heinlin et al. 2010; Kolb et al. 2008; Xinpei et al. 2009). The direct mechanism of inactivation of fungi using cold plasma is still not entirely clear. Scanning electron microscopy examination of plasma post-treated *Cordyceps bassiana* spores revealed dried, cracked and flattened propagules, indicating that cold plasma treatment may cause cell wall leakage and destruction, resulting in reduced cell viability (Lee et al. 2015). Similar results were achieved with cold plasma treatments of *Aspergillus* spp. (Dasan et al. 2017), which is of paramount importance since *Aspergillus* spp. are very common in MC floral parts, and can cause serious health complications in immunocompromised patients when mycotoxin-contaminated products are inhaled in large quantities (Gargani et al. 2011; Hamadeh et al. 1988; Ruchlemer et al. 2015). Even more intriguing is the reported ability of cold plasma treatment to reduce the presence of these toxins as well as pesticide residues (Misra et al. 2015; Sarangapani et al. 2016). While gamma, e-beam irradiation and cold plasma treatments appear promising for MC sterilization, there is a lack of evidence and knowledge regarding the efficacy of each of these methods, specifically in the treatment of harvested MC inflorescences, and their effect on the desired active chemical compounds.

In this research, we examined the efficacy of three sterilization methods: (i) gamma irradiation, (ii) beta irradiation (e-beam), and (iii) cold plasma sterilization, for reduction and elimination of fungal colony forming units (CFUs) in uninoculated and  artificially inoculated *B. cinerea* inflorescences, naturally infected commercial trimmed floral parts and inflorescences.

## Methods

### Plant and fungal samples

*Cannabis sativa* cultivar (BB 734) was grown in the Agriculture Research Organization (ARO) Volcani Center research facility (authorized by the Israeli Medical Cannabis Agency, IMCA, Ministry of Health, State of Israel) for this research. Uncharacterized cannabis seedlings were kindly provided by Dr. Moshe Flaishman, ARO that established the genetic source. Plants were propagated and five male flowers were used for pollination of 30 female flower plants. Seeds were collected from the harvested female flowers and sown for continuous breeding and selection for a range of parameters. The strain that was used in this study (BB 734) was derived from shoots of third generation mother plants. This strain is a “drug-type” cannabis with *Cannabis indica* characteristics.

Shoots were rooted under continuous 24 h light photoperiodic conditions of 880 LUX, for 1 week in a closed plastic planting container (80 × 40 × 50 cm) in a humid environment, and an additional week without the top cover. The rooted shoots were replanted in 0.2 L pots and transferred for vegetative propagation under photoperiodic conditions of 18 h light and 6 h dark of 3000 LUX, for 2 months. Plants were retransferred into 0.5 to 2 L pots and placed in a flowering induction chamber 4 × 3 m, for 80–90 days. The flowering chamber contained six 600 W high pressure sodium lamps (SunMaster, Twinsburg, Ohio, USA) with dual red and blue spectrum light, under photoperiodic conditions of 11 h light (50,000 LUX) and 13 h dark, until flowers were produced. Mature inflorescences, that were produced 80–90 days after floral induction, were used for sterilization experiments.

Two types of plant parts were used: (i) uninoculated (that included asymptomatic natural infections) mature inflorescences, (ii) artificially inoculated mature inflorescences with a culture of *Botrytis cinerea* originating from naturally infected cannabis flowers, isolated and characterized by morphological and molecular methods. It should be noted that “uninoculated” inflorescences from the ARO facility contained asymptomatic microbial infections comprised of a wide variety of different fungal species. *B. cinerae* was cultured for 2 weeks at 22 °C on 9 cm Petri plates containing potato dextrose agar (Difco, Franklin Lakes, New Jersey, USA) supplemented with 0.25 g/l chloramphenicol (PDAC) (Acros Organics, Geel, Belgium). After 14 days, spores were harvested from the plates with a sterile rod by adding a suspension of 10 ml sterile saline solution (NaCl 0.85 g/l, Tween 20, 100 μl/l). The conidia were filtered through four layers of sterile gauze and centrifuged (Heraeus, Franklin Lakes, New Jersey, USA) at 9000 RPM for 10 min at 4 °C. The pellet was resuspended in 20 ml fresh saline solution and adjusted to a concentration of 10^6^ spores/ml. The inoculum was sprayed till run-off on healthy mature cannabis plants that were subsequently covered by a plastic bag. After 5 days, the bags were removed and harvested flowers were dried in an Excalibur 3548/3948 digital oven (Sacramento, California, USA) at 35 °C for 12 h, then stored in a STATUS innovations vacuum pack (Metlika, Slovenia) at room temperature before experimentation. CFU’s of the microbial cultures from affected floral parts, before and after each sterilization treatment, were determined (see below).

### Quantification of fungal colonies

One g of each floral sample was inserted into a 10 ml sterile saline solution in 50 ml Falcon tubes, vortexed for 30 s and kept at room temperature for 10 min. Thereafter, serial dilutions were conducted and spread on PDAC plates that were maintained at room temperature (22°-25 °C) for 3–5 days, and developing CFU’s of total yeasts and mold (TYM) species were enumerated and characterized.

### Survey of CFU levels from commercial MC farms

In order to evaluate common CFU levels under commercial conditions, samples of MC inflorescences were collected from 4 different farms located in Israel. CFU levels were evaluated as described. Plating of each sample was conducted 3 times to achieve higher reproducibility. Variability in sampled inflorescences existed, as certain samples exhibited disease symptoms while others remained asymptomatic, and certain inflorescences were dry.

### Gamma and beta irradiation

Commercial cannabis samples were received from a number of commercial farms in Israel for irradiation treatments. Treatments comprising of e-beam (beta irradiation) and gamma irradiation were conducted at Sorvan Radiation Ltd., Soreq Nuclear Research Center, Israel. Gamma radiation was based on a ^60^Co isotope, doubly encapsulated in stainless steel source pencils type C-188, with radiation dosages of 7.5 and 8.37 KGy (KiloGray) in two consecutive experiments, respectively. E-beam radiation was created using an electron accelerator, with 15 kW (KWs) and an energy capacity of 5.25 megaelectronvolt (MeV). The radiation dosages were 4.18, 8.2 and 10.26 KGy, and 4.06, 8.5, and 10.26 KGy in two consecutive experiments, respectively.

### Cold plasma irradiation

Cold plasma treatment was conducted using a prototype created by NovaGreen company, (Kibbutz Megiddo, Israel). The gas in this treatment was low pressure air with the addition of H_2_O_2_ liquid at a concentration of 35% (Chen Shmuel Chemicals Ltd., Haifa, Israel). A vacuum chamber was generated using an Edwards i10 dry pump to eliminate possible oil contamination that may have occurred during wet pump usage. Although the H_2_O_2_ liquid had no direct contact with the MC, it affects the gaseous environment and generates a highly reactive plasma with elevated concentrations of oxygen species. An RF generator at a voltage of 6 kV generated the plasma and exposure periods lasted for 2.5, 5.0 and 10 min for each experiment.

### Sampling procedures and experimental design

Two sample types [uninoculated (that included asymptomatic infections) and artificially inoculated *Botrytis cinerea*] of noncommercial plant material were obtained from the ARO Volcani Center facilities and divided into bags containing 5 g MC floral parts each (total of 20 g per sample type). Artificially inoculated *Botrytis cinerea* and uninoculated samples were treated with beta and gamma radiation at Sorvan facility. A 5 g non-irradiated sample of each floral MC type served as a control. After irradiation treatments, four and three biological repeats (from two consecutive experiments, respectively) were removed from each bag and CFU’s were determined, as described.

Irradiation treatments of naturally infected commercial plant material including (i) dried and packed floral parts, and (ii) dried and packed trimmed leaves were assessed for efficacy of treatments by determining CFU counts. Inflorescences were naturally infected indicating that CFUs from these inflorescences were comprised of a wide variety of fungal species. Each product contained two 500 g vacuum-sealed bags that were treated with e-beam irradiation at Sorvan nuclear facility. A 5 g sample that was removed from each bag before the irradiation treatments served as a control. To determine efficacy of e-beam irradiation treatments at different locations in the bag, six samples of 5 g each were removed after treatments from different locations of each bag: from the upper right corner, upper left corner, lower right corner, lower left corner, upper middle area and lower middle area, and CFU’s were determined as described.

Cold plasma treatment was conducted on noncommercial floral material. The experimental design was identical to that described for the noncommercial irradiation experiments. Floral parts were placed on the electrode and H_2_O_2_ was injected around the perimeter. Each treatment comprised of 8 min of vacuum and different plasma exposure periods described. An untreated sample served as control.

### ED_50_ calculation

In order to determine the effective radiation dosage for eliminating 50% (ED_50_) CFUs, a response curve with *R*^*2*^ > 0.95 was produced for each treatment. CFU levels in the controls of each treatment were calculated as the 100%. This value divided by two was used as the Y value in the response curve formula of each treatment, and served as the radiation dosage required for reducing CFU levels by 50% (ED_50_). All other ED values were calculated using the same method.

## Results

### CFU survey of inflorescences from commercial farms

**Fig. 1:**
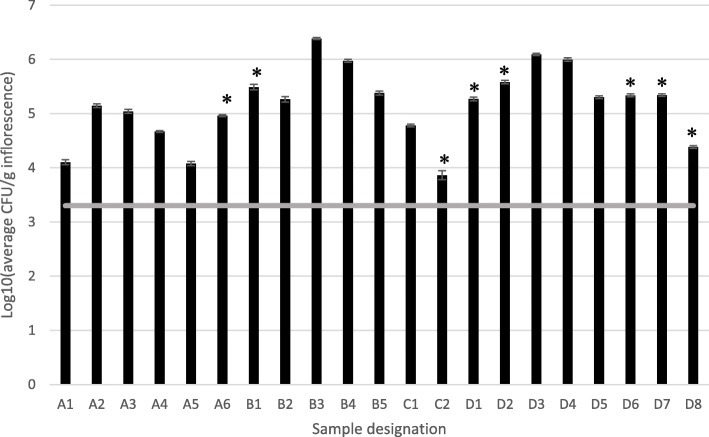
CFU levels [log10(CFU/g inflorescence)] detected from four farms designated A, B, C and D. Consecutive numbers after each individual farm indicate different samples of inflorescences taken from that farm. Samples D1 and D2 represent dried inflorescences. Asterisks indicate MC inflorescence that were asymptomatic, all other samples exhibited varying degrees of disease symptoms. Bars represent SE of the mean of 3 replicates per sample. The gray line represents maximum levels of total yeasts and molds permitted according to protocols of the IMCA for commercial MC inflorescences (2000 CFU/g)

The initial CFU survey that was conducted on 21 samples indicated that in all four tested commercial farms levels of TYM fungal contamination exceeded the maximum CFU values of 2000 yeasts and molds per g inflorescence permitted by the IMCA (Fig. 1); some samples exceeded this level by as much as 3.08 log-fold CFU/g inflorescences. Morphological identification of the CFUs indicated an abundance of the following fungal species; *Alternaria* spp., *Aspergillus* spp., *Botrytis cinerea*, *Fusarium* spp., and *Penicillium* spp.

### Irradiation treatments

#### Gamma irradiation of noncommercial MC inflorescences

Gamma irradiation treatments caused a considerable reduction in TYM CFU levels in noncommercial MC inflorescences. CFU levels were reduced in the uninoculated MC inflorescence samples from 6.16 ± 0.2 and from 6.04 ± 0.08 to 0 log CFU/g inflorescence in the first (7.5 KGy irradiation dosage) and second (8.37 KGy) experiments, respectively.

In artificially inoculated *Botrytis cinerea* MC inflorescences, gamma irradiation treatments resulted in a reduction of CFU levels from 8.05 ± 0.12 and 7.7 ± 0.11 to 1.88 ± 0.96 and 3.02 ± 0.11 log CFU/g inflorescence, in consequent experiments respectively, a respective reduction of 6- and 4.5 log-fold. Peak temperatures measured during irradiation treatments reached 32.5 and 30.0 °C in the first and second experiments, respectively.

#### E-beam (beta irradiation)

##### E-beam treatments of noncommercial material

**Fig. 2:**
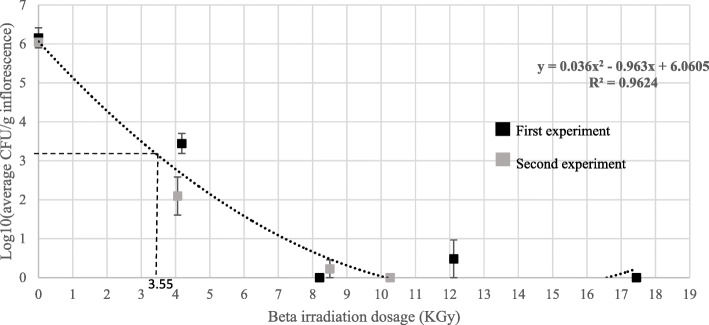
CFU levels [log10(CFU/g inflorescence)] of uninoculated MC inflorescences, exposed to different e-beam irradiation dosages in two experiments. Bars represent SE of the mean of 9 replicates per sample. A value of 3.55 KGy was calculated, according to the polynominal formula (dotted line), to reduce CFUs by 50% (ED_50_), represented by the dashed line

E-beam treatments at 10.26 KGy of noncommercial MC floral parts were very effective in completely eliminating contamination of uninoculated inflorescences from 6.16 ± 0.26 and 6.04 ± 0.08 to 0 log CFU/g inflorescence in two consequent experiments, respectively (Fig. 2).

**Fig. 3:**
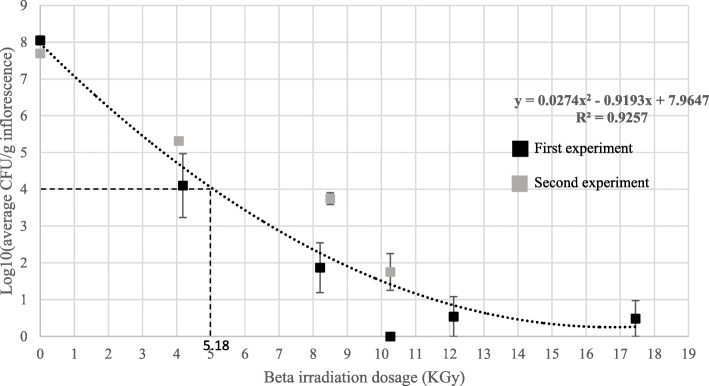
CFU levels, [log10(CFU/g inflorescence)] of artificially inoculated *Botrytis cinerea* MC inflorecences, exposed to different e-beam irradiation dosages in two experiments. Bars represent SE of the mean of 9 replicates per sample. A value of 5.18 KGy was calculated, according to the polynominal formula (dotted line) to reduce CFUs by 50% (ED_50_), represented by the dashed line

**Fig. 4:**
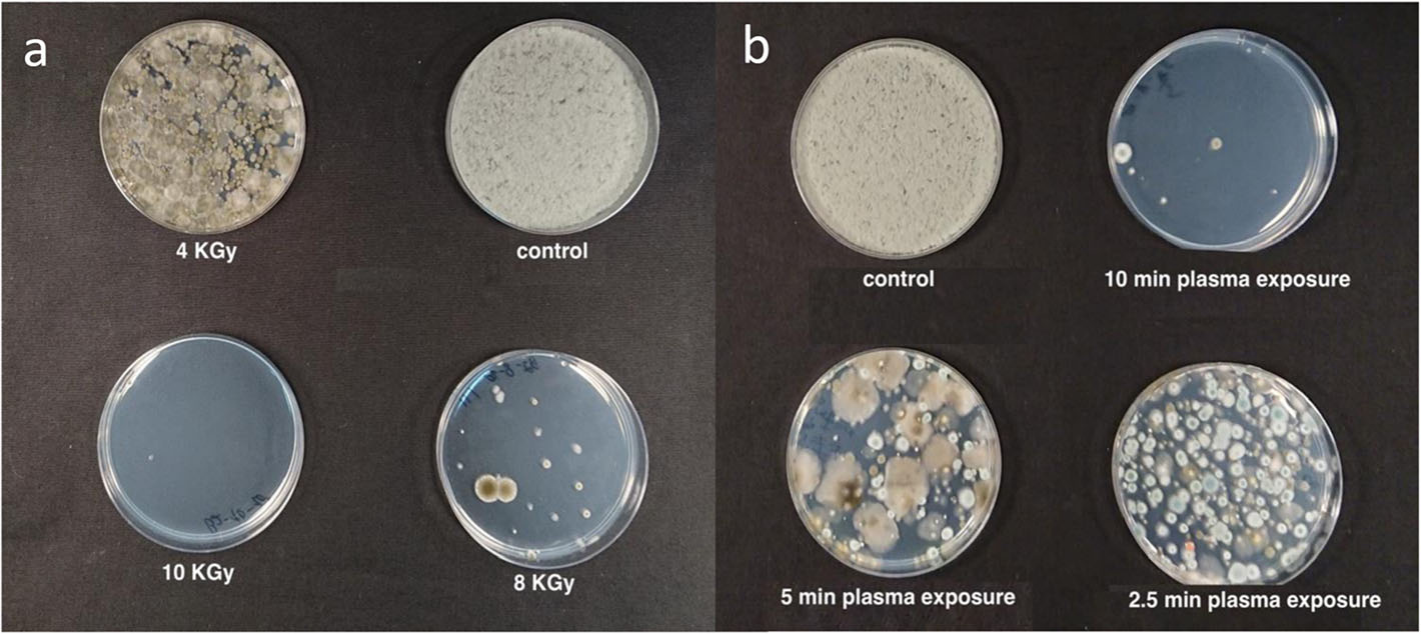
Fungal CFUs from artificially inoculated *Botrytis cinerea* inflorescences after: **a** e-beam irradiation (4, 8, 10 KGy and untreated control), and **b** cold plasma (2.5, 5, 10 min exposure and untreated control) treatments

A similar pattern was found when noncommercial, artificially inoculated *Botrytis cinerea* MC inflorescences were treated under the same conditions, (Figs. 3 and 4a). E-beam irradiation reduced CFU levels in two consecutive experiments to 0 and 1.75 ± 0.5 log CFU/g inflorescence, respectively (Fig. 3). In both experiments, peak temperatures during radiation were less than 27 °C.

The effective dosages calculated for reduction of percent population of CFUs for e-beam treatments in artificially inoculated *Botrytis cinerea* and uninoculated MC inflorescences are shown in Table 1.

**Table 1:** Effective dosage of e-beam treatments for reducing percent CFU populations in both infected and artificially inoculated *Botrytis cinerea* MC inflorescences

Effective dosage (ED)(%)	Irradiation dosage (KGy) of uninoculated inflorescences^a^	Irradiation dosage (KGy) of *B. cinerea* inoculated inflorescences^b^
90	8.1	12.4
70	5.5	8
50	3.6	5.2
10	0.6	1

^a^Calculated using the polynomial formula in Fig. 2

^b^Calculated using the polynomial formula in Fig. 3

While the ED values in the uninoculated MC inflorescences were considerably lower than those for the artificially inoculated *Botrytis cinerea* inflorescences, it is worth mentioning that in the inoculated inflorescences (Fig. 3) initial CFU levels were 100-fold higher than those of the uninoculated inflorescences (Fig. 2).

##### E-beam treatments of commercial material

**Fig. 5:**
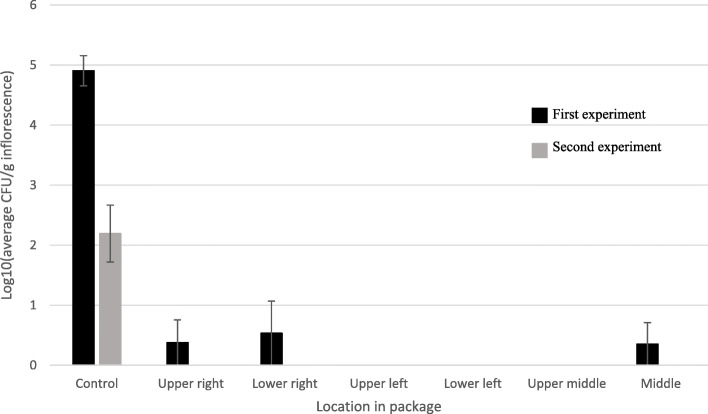
CFU levels [log10(CFU/g inflorescence)] in naturally infected commercial MC inflorescence before (control) and after e-beam treatments in two experiments. Post-treatment samples were taken from different locations of vacuum-sealed packages. Bars represent SE of 3 replicates per sample per location

Beta-irradiation of commercial material significantly reduced and eliminated CFUs in MC inflorescences at all locations in the vacuum-sealed packages. In the first experiment, e-beam reduced CFU levels from 4.9±0.25 to 0 log CFU/g inflorescence, compared to the untreated control. (Fig. 5). Likewise, in the second experiment, CFUs were reduced to undetected levels at all sampled locations in the package (Fig. 5).

**Fig. 6:**
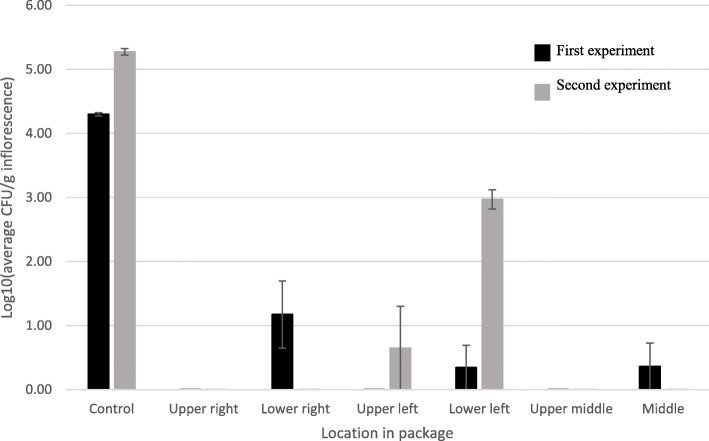
CFU levels, [log10(CFU/g inflorescence)] in naturally infected commercial MC trimmed leaves before (control) and after e-beam treatments in two experiments. Post-treatment samples were taken from different locations of vacuum-sealed packages. Bars represent SE of 3 replicates per sample per location

The results of irradiation of commercial MC trimmed leaves were more varied (Fig. 6). In the first experiment, no viable CFUs were detected from three of the sampled locations, although CFU levels were significantly reduced in three of the other locations, compared to the untreated controls (Fig. 6). In the second experiment, even though initial CFU levels were higher, no CFUs were detected from four sampled locations after the treatment (Fig. 6).

#### Cold plasma treatments of noncommercial material

**Fig. 7:**
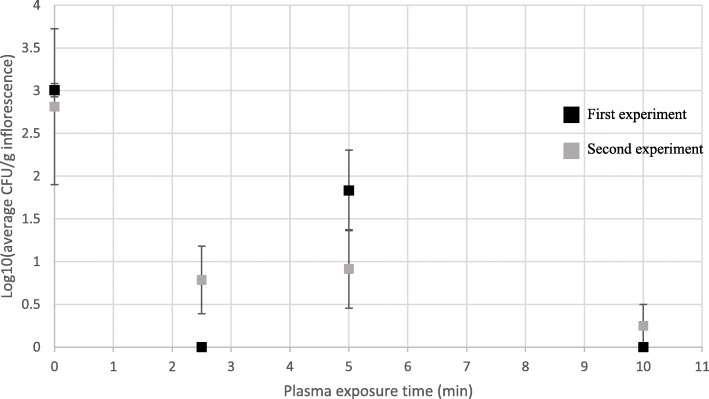
CFU levels [log10(CFU/g inflorescence)] of uninoculated MC inflorescences exposed to different cold plasma treatments. Bars represent SE of the mean of 9 replicates per sample

Cold plasma treatments resulted in a reduction in CFU levels of uninoculated inflorescences, according to exposure times (Fig. 7). After 2.5 min of plasma exposure, CFU levels were reduced from 3.01 ± 0.08 and 2.81 ± 0.99 log CFU/g inflorescence to 0 and 0.79 ± 0.39 log CFU/g inflorescence, in the first and second experiments, respectively (Fig. 7). However, after 5 min exposure the detected CFU levels were 1.83 ± 0.47 and 0.92 ± 0.46 log CFU/g inflorescence, and after 10 min of exposure, CFU levels were reduced to 0 and 0.25 ± 0.25 log CFU/g inflorescence, in the respective consecutive experiments, a reduction of approximately 3-log-fold in detected CFU levels. In an experiment performed with heavily infected uninoculated inflorescences, a reduction of approximately 6 logs was recorded, to below 10 CFU/g inflorescence after 12 min of plasma exposure (data not shown).

**Fig. 8:**
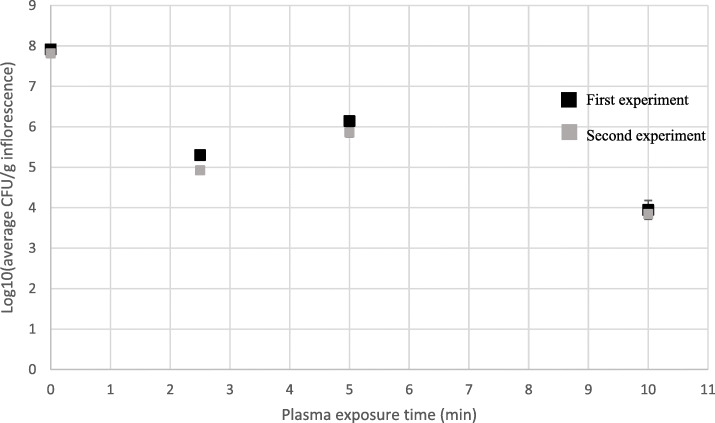
CFU levels, log10(CFU/g inflorescence), of *Botrytis cinerea-*inoculated MC inflorescences exposed to different cold plasma tretments. Bars represent SE of the mean of 9 replicates per sample

A similar pattern was observed in artificially inoculated *Botrytis cinerea* MC inflorescences treated with cold plasma (Figs. 4b and 8). After 2.5 min of plasma exposure, CFU levels were reduced by approximately 3-log-fold, by 2-log-fold after 5 min exposure, and 4-log-fold after 10 min exposure (Fig. 8).

## Discussion

The use of medical cannabis (MC) has increased tremendously in the last decade (Ruchlemer et al. 2015). One of the main reasons for the rising popularity and interest in MC are the therapeutic qualities of this plant (Ben Amar 2006; Cascio et al. 2017; Russo 2011; Sirikantaramas and Taura 2017). With the increase in use, both for recreational and therapeutic means, reports are starting to accumulate concerning the threat of microbial presence in MC inflorescences and the harmful potential to cannabis consumers, especially in immunocompromised patients (Gargani et al. 2011; Hamadeh et al. 1988; McPartland and McKernan 2017; Ruchlemer et al. 2015). The extent of MC inflorescence infections by fungal CFUs was determined in our initial survey of commercial farms, indicating that without sterilization treatments, levels of CFUs were extremely high, above that permitted by the IMCA in all tested sites, disregarding the sample condition; dried or un-dried, and with or without visible disease symptoms (Fig. 1). Since it is not feasible to cultivate commercial MC under a sterile environment there is an acute need for postharvest MC inflorescence sterilization before usage (Hazekamp 2016).

There have been many reports regarding the efficacy of different non-thermal treatments for food and herb sterilization, especially the utilization of gamma irradiation (Guerreiro et al. 2016; Jeong et al. 2015; Sádecká 2007). Alternatively, e-beam (beta irradiation) and cold plasma, are effective for food sterilization and are safe for human consumption (Jeong et al. 2015; Misra et al. 2019; Moreno et al. 2007; Van Impe et al. 2018). Even though these methods have been applied or suggested for decontaminating MC inflorescences to safeguard its use by immunocompromised patients, there is a lack of knowledge in regards to their efficacy in eliminating deleterious microorganisms.

In this research, we examined the effect of gamma irradiation, e-beam and cold plasma treatments, on the reduction of CFU contamination in artificially inoculated and naturally infected MC inflorescences and trimmed leaves (Table 2). Gamma irradiation was very effective in reducing the CFU levels by approximately 6-log-fold CFU/g inflorescences, at a minimal dosage of 7.5 KGy. Similarly, in the Netherlands, MC is sterilized using ^60^Co gamma irradiation at a dosage of 10 KGy, which is well below the authorized dosage of 30 KGy, permitted by the FDA for irradiation of aromatic herbs and spices (Sádecká 2007). Likewise, in a recent report, ^60^Co gamma irradiation was used to sterilize cherry tomatoes with a radiation dosage of 5.7 KGy that reduced CFU levels from 2.2 log CFU/g to nearly zero, an inactivation efficacy of 99.8% (Guerreiro et al. 2016). In spite of its effectiveness, gamma irradiation remains an expensive sterilization method requiring the usage of radioactive isotopes, specialized equipment and facilities. In contrast, e-beam does not require the use of radioactive isotopes and as such, is considerably more environmentally friendly (Leonhardt 1990). Moreover, in this research we found that e-beam treatments were very effective in eliminating CFUs from infected MC inflorescences applied at low temperatures, below 27 °C, that do not detrimentally affect the active ingredients of MC. In fact, this method is so effective that at a radiation dosage of 10.26 KGy, CFU levels were reduced from 6 log CFU/g inflorescence to 0 (Fig. 2). A similar reduction in CFUs was observed with artificially inoculated inflorescences (Fig. 3).

**Table 2:** Effect of radiation and sterilization methods on fungal contamination of cannabis plant samples

Treatment	Experiment number	Sample type	Average CFU before treatment [log 10 CFU/g inflorescence]	Radiation dose [kGy]	Plasma exposure time [min]	Average CFU after treatment [log 10 CFU/g inflorescence]
Gamma irradiation	1	Uninoculated inflorescence	6.16 ± 0.26	7.5	–	0
	1	*Botrytis cinerea-*inoculated inflorescence	8.05 ± 0.12	7.5	–	1.88 ± 0.96
	2	Uninoculated inflorescence	6.04 ± 0.08	8.37	–	0
	2	*Botrytis cinerea-*inoculated inflorescence	7.7 ± 0.11	8.37	–	3.02 ± 0.11
E-beam	1	Uninoculated inflorescence	6.16 ± 0.26	10.26	–	0
	1	*Botrytis cinerea-*inoculated inflorescence	8.05 ± 0.12	10.26	–	0
	2	Uninoculated inflorescence	6.04 ± 0.08	10.26	–	0
	2	*Botrytis cinerea-*inoculated inflorescence	7.7 ± 0.11	10.26	–	1.75 ± 0.5
	1	Naturally infected commercial inflorescence	5 ± 0.25	11.99	–	0.211 ± 0.1
	2	Naturally infected commercial inflorescence	2.2 ± 0.47	11.99	–	0
	1	Naturally infected commercial trimmed leaves	4.3 ± 0.03	11.99	–	0.314 ± 0.095
	2	Naturally infected commercial trimmed leaves	5.27 ± 0.05	11.99	–	0.6 ± 0.48
Cold plasma	1	Uninoculated inflorescence	3 ± 0.08	–	10	0
	2	Uninoculated inflorescence	2.81 ± 0.91	–	10	0.25 ± 0.25
	2	*Botrytis cinerea-*inoculated inflorescence	7.82 ± 0.04	–	10	3.84 ± 0.08
Cold plasma with changing vacuum time^a^	1	Uninoculated inflorescence	6.6 ± 0.35	–	12.5 min + 10 min vacuum	0.25 ± 0.25
	1	*Botrytis cinerea-*inoculated inflorescence	6.88 ± 0.11	–	10 min + 8 min vacuum	4.05 ± 0.31

*Abbreviations*: *CFU* colony forming units, *kGy* kiloGray

^a^in the cold plasma treatment with changing vacuum time, only one experiment was conducted

ED values are an important and useful tool as they indicate the efficacy of treatments and also allow for comparison between different sterilization systems; in general lower ED values represent a higher acute toxicity (Lorke 1983). ED values are extremely relevant in this study since they indicate a measurable value for the efficacy of the treatments. The ED values calculated for e-beam irradiation (Table 1) were also very promising; e.g. ED_10_, the radiation dosage required to reduce 10% CFU populations of uninoculated and artificially inoculated inflorescences were calculated as 0.6 KGy and 1 KGy, respectively. In comparison, the ED_10_ value for inactivation of enteric viruses e.g. poliovirus Type 1 on cantaloupe surfaces using e-beam was 4.76 KGy (Shurong et al. 2006). Similarly, e-beam treatment of red pepper powder at a dosage of 3 KGy, reduced CFU levels of total yeasts and molds from 6.62 to 3.71 log CFU/g (Kyung et al. 2019). In our research, we found that a dosage of 12.4 KGy resulted in 90% reduction in CFU levels in artificially inoculated *B. cinerea* MC inflorescence (Table 1). Although 12.4 KGy is considered a rather high radiation dosage, it is important to note that in this experiment the initial CFU levels were very high (more than 7.7 ± 0.11 log CFU/g). Accordingly, initial CFU levels in commercial MC were constantly much lower, measuring below 5 ± 0.25 CFU/g. This indicates that irradiation values of 8.1 KGy, resulting in a reduction of 90% of uninoculated MC inflorescences are very reasonable considering that the irradiation dosages are much lower than the maximum limit of 30 KGy, according to that authorized by the FDA for irradiation of dry herbs (Sádecká 2007).

Cold plasma treatment of MC inflorescences was also found to be effective for elimination of fungal propagules in this study. After 10 min of plasma exposure, CFU levels in uninoculated MC inflorescences were reduced by approximately 3 log-fold CFU/g. It is important to note that initial recorded infection levels in these experiments were approximately 3 log CFU/g indicating that given a higher infection level, an exposure to 10 min plasma treatment may have resulted in an even greater CFU reduction (Fig. 7). In an additional experiment (data not shown) a cold plasma treatment of 8 min vacuum time followed by 12 min of plasma exposure resulted in CFU reduction of approximately 6 log CFU/g of uninoculated MC inflorescences. Accordingly, in artificially inoculated *B. cinerea* MC inflorescences the results were improved with a reduction of approximately 4 log CFU/g, after 10 min of plasma exposure (Fig. 8). Similarly, cold plasma treatments conducted in Shaare Zedek Medical Center Israel, resulted in complete sterilization of MC inflorescences (Ruchlemer et al. 2015). In that research, autoclaving and ethylene gas sterilization were found to be as effective for MC inflorescence sterilization, although the two latter methods resulted in a greater reduction of Δ^9^-THC, which is one of the major phytocannabinoids in MC. Cold plasma was also found useful in decontamination of wheat seeds, resulting in complete inactivation of *Fusarium nivale*-artificially inoculated seeds after as little as 90 s treatment (Zahoranová et al. 2016). An increase in CFU counts after 5 min of plasma exposure compared to that after 2.5 min was recorded in our experiments, which is still unclear (Figs. 7 and 8) and will require further research. In various studies, cold plasma was reported to degrade both mycotoxins and pesticides in in vitro experiments (Ten Bosch et al. 2017; Misra et al. 2011; Sarangapani et al. 2016). Mycotoxins such as aflatoxins, zearalenone and fumonisins are secondary metabolites produced by certain fungi such as *Aspergillus* and *Fusarium* spp. that are ubiquitous in MC inflorescences and are known to cause health risks to humans and mammals (McPartland et al. 2017; Misra et al. 2019). Thus, cold plasma treatments may be even more beneficial by not only reducing CFU counts but also by reducing levels of mycotoxins (Ten Bosch et al. 2017; Misra et al. 2019).

Another important aspect when dealing with MC are the active compounds, comprised mainly of different phytocannabinoids and terpenoids. These compounds, especially phytocannabinoids, are responsible for MC therapeutic effects and currently more than 100 different phytocannabinoids have been identified (Cascio et al. 2017; Russo 2011). In recent years, there is a growing understanding that some of the MC therapeutic affects are a result of synergism among the bioactive compounds in certain MC lines and cultivars (Russo 2011). Thus, it is crucial to decontaminate MC inflorescences using a method that causes the least damage to the profiles of these active compounds.

## Conclusions

In this research, we tested 3 different methods for MC inflorescence sterilization, all three proving to be effective (Table 2). Gamma irradiation was very effective in reducing total yeast and mold (TYM) CFU levels but is not environmentally friendly and requires a nuclear facility. On the other hand, e-beam (beta) irradiation does not require the use of radioactive isotopes and is much faster and easier to apply, possessing high efficacy in reducing TYM CFUs, achieving maximum CFU reduction of approximately 8-log-fold (Table 2). Cold plasma was also effective in reducing TYM CFU levels, reaching maximum CFU reduction of approximately 6-log-fold (Table 2). Assessing fungicide and mycotoxin degradation effects of cold plasma and e-beam in MC inflorescences in vivo, and also the effect of both e-beam and cold plasma treatments on active compounds in MC, will require further, extensive research. However, both of these methods appear to possess the potential in producing clean, safe and healthy MC products.

## Data Availability

All data generated or analyzed during this study are included in this published article.

## Abbreviations

^60^Co: 60 cobalt isotope
ARO: Agriculture Research Organization
CFU: Colony forming unit
E-BEAM: Electron beam
ED: Effective dosage
IMCA: Israeli Medical Cannabis Agency
KGy: KiloGray
KWs: Kilowatts
MC: Medical Cannabis
MeV: Megaelectronvolt
PDAC: Potato dextrose agar and chloramphenicol
ROS: Reactive oxygen species
TYM: Total yeast and mold
